# Energy Drinks as the Legal Cocaine? A Comparative Review of Cardiac Physiopathological and Histopathological Patterns

**DOI:** 10.1007/s12012-025-10069-5

**Published:** 2025-11-21

**Authors:** Alessandro Ghamlouch, Nicola Di Fazio, Maura Racciatti, Fabio Del Duca, Biancamaria Treves, Gaia De Angelis, Alessandra De Matteis, Aniello Maiese, Paola Frati

**Affiliations:** 1 Department of Anatomical, Histological, Forensic and Orthopaedic Sciences, Sapienza University of Rome, Viale Regina Elena 336, 00161 Rome, Italy; 2 Department of Biomedicine and Prevention, University of Rome “Tor Vergata”, Via Montpellier 1, 00133 Rome, Italy; 3 Department of Life Sciences, Health and Health Professions, Link Campus University, Rome, Italy

**Keywords:** Energy drinks, Cocaine, Cardiovascular toxicity, Cardiac histopathology, Cardiac pathophysiology

## Abstract

**Abstract:**

Energy drinks (EDs), widely consumed for their stimulant effects, typically contain caffeine alongside taurine, guarana, and other bioactive compounds. While generally regarded as safe, growing evidence links chronic EDs consumption to significant cardiovascular risks. Caffeine, the primary active ingredient, acts through adenosine receptor antagonism and increased calcium release, potentially provoking arrhythmias and myocardial stress. Taurine and other additives further influence cardiac excitability and contractility. This systematic review, conducted under PRISMA 2020 guidelines, investigated the cardiac histopathological consequences of chronic EDs use. A literature search spanning 2021 to March 2025 across PubMed, Google Scholar, and Scopus identified studies reporting EDs-related cardiac effects. Data extraction and analysis revealed consistent associations with QTc prolongation, atrial and ventricular arrhythmias, myocardial infarction, Takotsubo cardiomyopathy, and hypertensive episodes—even in young, healthy individuals. Animal studies support these findings, showing myocardial necrosis, myofiber disarray, mitochondrial damage, and inflammation, particularly when EDs are combined with alcohol. Notably, similarities between EDs and cocaine emerged, including shared mechanisms involving ion channel blockade, sympathetic overactivation, vasoconstriction, and prothrombotic states. Chronic use of either substance can result in structural heart damage and remodelling. Although EDs and cocaine differ in legal status and potency, their overlapping cardiovascular effects warrant greater clinical awareness and public education. Excessive EDs consumption poses a real cardiotoxic risk, especially in vulnerable populations, underscoring the need for further human research and potential regulatory consideration.

**Graphical Abstract:**

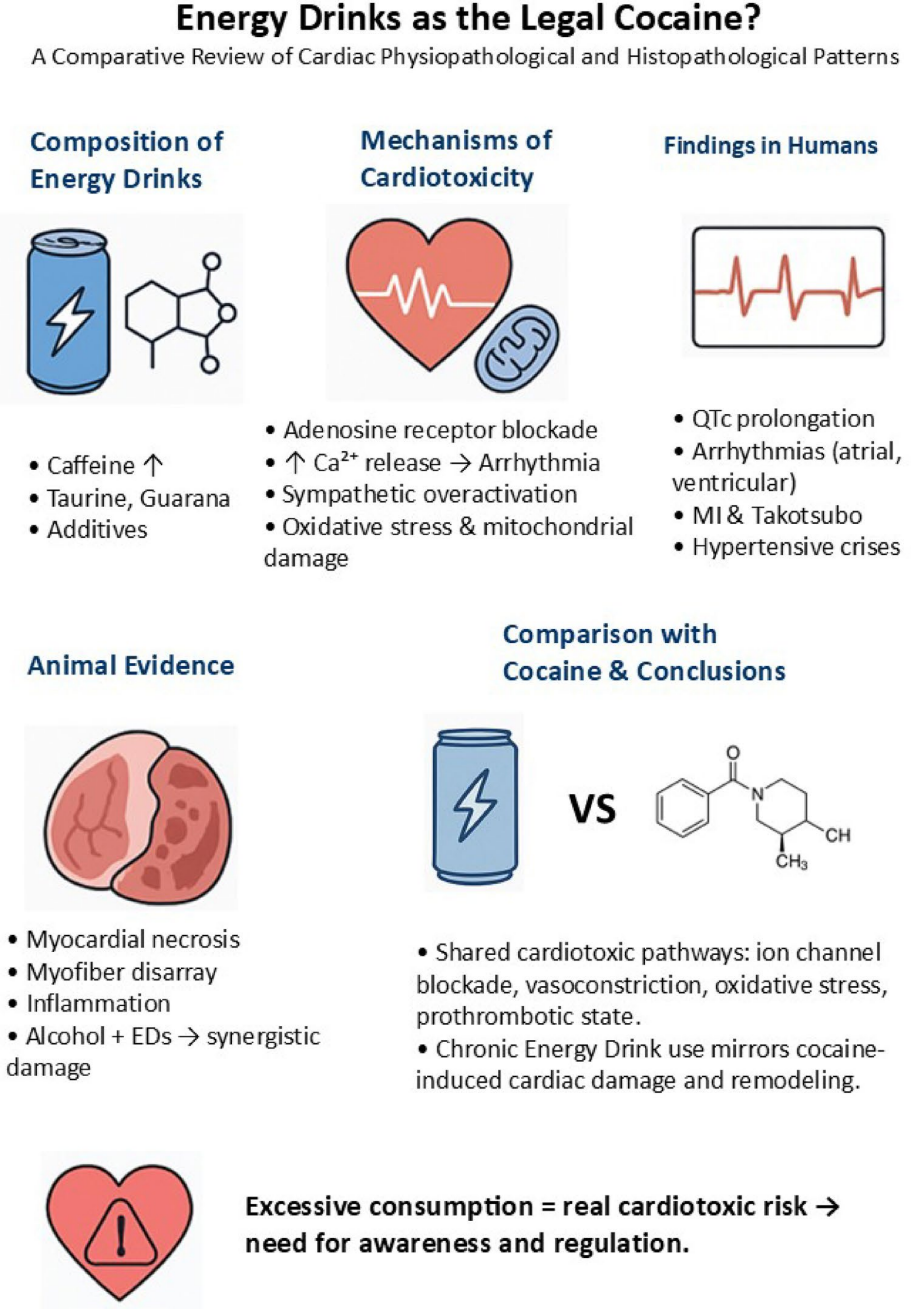

## Introduction

Energy drinks (EDs), according with the Food and Drug Administration (FDA), are “a class of products in liquid form that typically contains caffeine, with or without other added ingredients” [1]. This beverage category has garnered significant attention, as demonstrated by its widespread consumption across diverse demographic groups, including adolescents, workers, students, professional and amateur athletes, and nightlife participants, irrespective of their health risk profiles [2].

Many studies analysed the cardiovascular impact of EDs showing myocardial effects, in particular on blood pressure and heart rate.

In order to understand these effects of EDs it’s important to evaluate the main ingredients contained in the drink: **Caffeine** (a xanthine alkaloid with stimulating effects on the central nervous system and myocardium); **Taurine** (an amino acid involved in osmoregulation, neurotransmission, and muscle contractility); Guarana (a natural source of caffeine, theobromine, and theophylline); Ginseng (an adaptogen with vasodilatory and stimulant properties); B vitamins (coenzymes involved in energy metabolism); simple sugars (a rapid source of energy, but associated with cardiometabolic risk), carnitine, choline, vitamin C, vitamin A (beta carotene), vitamin D, electrolytes (sodium, potassium, magnesium, and calcium), tyrosine, and l-theanine, with prevalence for each ingredient ranging from 1.3 to 100% [3].

Evaluating these cardiac effects and the similarity with the **cocaine’s** action it’s important to consider the physio pathological and morphological pattern of the cardiac tissue after cocaine’s abuse.

In particular, cocaine is a naturally derived narcotic substance extracted from the *Erythroxylum coca* plant. It is a stimulant that affects cardiovascular physiology by influencing heart rate, blood pressure, and the overall functioning of the heart. Initially, cocaine was used therapeutically as a local anaesthetic. However, today it is primarily abused for its euphoric and stimulant effects. Cocaine exerts its action through multiple molecular mechanisms that induce physiological alterations, which in turn lead to cardiac pathologies such as arrhythmias and myocardial ischemia. These conditions are responsible for the high mortality rate among both acute and chronic users. The cardiotoxic effects of cocaine are mediated by various pathophysiological mechanisms, including sympathetic nervous system hyperactivation, coronary vasospasm, arrhythmogenesis, thrombogenesis, and direct myocardial cytotoxicity [4, 5].

### Molecular Mechanisms of Cardiotoxicity (EDs Ingredients and Cocaine)

Considering the mechanism of action of **caffeine**, its cardiovascular stimulation is evident. It plays a role in stimulating the release of Ca²⁺ from the sarcoplasmic reticulum through its interaction with ryanodine receptors and its ability to inhibit phosphodiesterase activity. Moreover, caffeine influences blood pressure by increasing vascular resistance and blocking adenosine receptors, leading to a contractile effect [6].

In particular, caffeine affects multiple systems in the body, including the cardiovascular, respiratory, and both somatic and autonomic nervous systems [7–9]. Its molecular effects are mediated through four key mechanisms: (a) antagonism of adenosine receptors, (b) inhibition of phosphodiesterase, (c) stimulation of intracellular calcium release, and (d) antagonism of benzodiazepine receptors [9, 10]. The central nervous system stimulation is primarily due to the blockade of adenosine receptors [9, 11]. Phosphodiesterase inhibition leads to an increase in intracellular cyclic AMP, promoting lipolysis and glycolysis, which results in the release of free fatty acids and glucose into the bloodstream, providing energy [9, 10]. This mechanism also enhances cardiac stimulation by mimicking sympathetic nervous system activation. Stimulation of beta-1 receptors by adrenaline and noradrenaline activates adenylate cyclase, converting ATP into cyclic AMP. Caffeine slows the degradation of cAMP by inhibiting phosphodiesterase, amplifying the cardiac response. This results in positive inotropic, chronotropic, and batmotropic effects, which can trigger tachyarrhythmias even in healthy individuals or those predisposed (e.g., Wolff-Parkinson-White syndrome, dual AV node pathways, or post-myocarditis scarring) [9]. Sympathetic activation by caffeine also causes bronchodilation and increased respiratory rate, typical of the “fight or flight” response [8–10]. At high doses, caffeine further stimulates the release of calcium from intracellular stores like the sarcoplasmic reticulum, especially affecting cardiac and skeletal muscle cells. This can increase cellular contractility and, in genetically predisposed individuals (e.g., those with ryanodine receptor or calsequestrin mutations), may provoke calcium-dependent arrhythmias [12, 13]. At very high concentrations, caffeine can also inhibit benzodiazepine receptors, contributing to heightened central nervous system stimulation [8, 11].

Furthermore, also the **taurine** presents a role in the regulation of Regulation of Intracellular Calcium (taurine helps maintain intracellular calcium balance). Additionally, taurine can influence the duration of the cardiac action potential and the recovery phase (restoration of the membrane), improving the synchronization of heartbeats. Taurine appears to modulate the activity of Beta-adrenergic receptors, helping to improve cardiac function during stress or exertion without overloading the heart. Taurine also has a positive effect on cardiac contractility, enhancing the heart’s pumping capacity [14].

These substances exert multiple cardiovascular effects through different molecular mechanisms. In particular, caffeine acts as an antagonist of adenosine A1 and A2a receptors. This antagonistic action removes the natural inhibition that these receptors exert on adrenergic stimulation. As a result, intracellular cyclic AMP (cAMP) levels increase, leading to the activation of β1-adrenergic receptors and sympathetic stimulation, with a positive chronotropic and inotropic effect on myocardial cells [15].

Also, one of the primary mechanisms of **cocaine** involves adrenergic hyperactivation, which in turn generates hemodynamic stress. In fact, cocaine blocks the reuptake of catecholamines (dopamine, norepinephrine, and epinephrine) at presynaptic terminals. This leads to an accumulation of catecholamines at the synaptic level, resulting in prolonged stimulation of adrenergic receptors. This cascade causes an increase in heart rate, myocardial contractility, and blood pressure. The sudden rise in afterload, along with increased myocardial oxygen demand, exposes the heart to a high risk of ischemia - even in young individuals without pre-existing coronary artery disease [4].

The second major effect produced by cocaine involves endothelial dysfunction, which leads to coronary vasospasm. Nitric oxide (NO) acts directly on vascular smooth muscle, causing endothelium-dependent vasodilation. Since cocaine inhibits nitric oxide synthesis, this results in a direct effect on vascular smooth muscle—namely, a potent vasoconstrictive action at the level of the coronary arteries. Additionally, cocaine induces the expression of endothelin-1, a peptide with strong vasoconstrictive properties [16, 17].

The result of all this is the acute narrowing, or vasospasm, of the coronary arteries, leading to ischemia, angina pectoris, and myocardial infarction. This can occur even in the absence of atherosclerotic lesions [18].

The third pathological mechanism to consider when discussing cocaine and the heart is the alteration of cardiac electrical conduction, resulting in the development of arrhythmias.

Furthermore, cocaine also affects platelet activation, leading to an increased risk of thrombosis. It enhances the production of thromboxane A2 and reduces endogenous fibrinolytic activity, thereby increasing platelet activity.

Considering all these aspects, the typical morphologic lesion in cocaine-related death is an expression of catecholamine myotoxicity, characterized by **contraction band necrosis (CBN)** and a distinctive pattern of **microfocal myocardial necrosis**, which is pathognomonic of catecholamine myotoxicity. Additionally, **patchy myocardial fibrosis** may be present. In the early stages of myocardial remodelling, **cardiomyocytes typically appear thickened**,** with “boxcar” (squared-off) nuclei**. Another characteristic finding is **myofiber disarray** [6, 19].

**Fig. 1:**
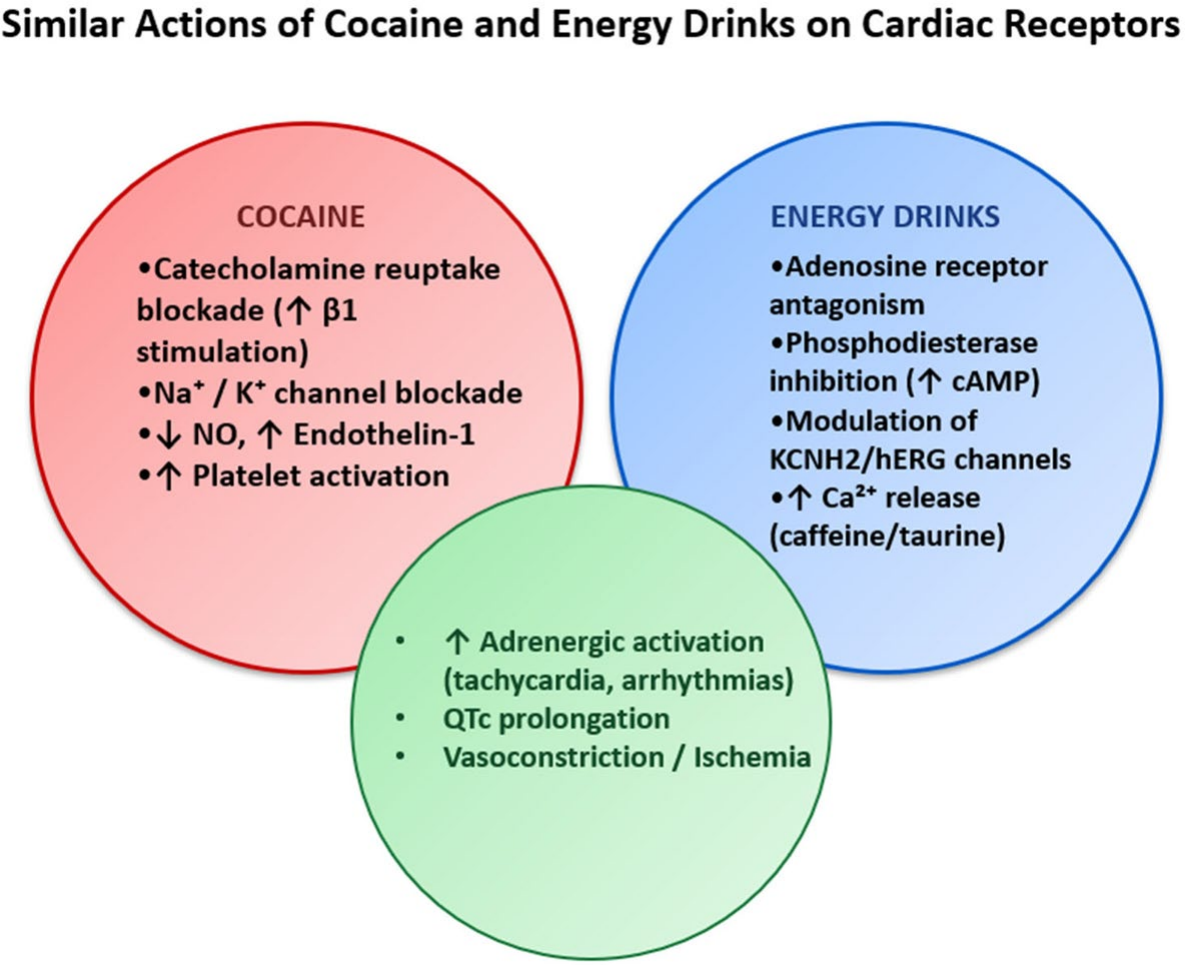
Comparison between cocaine and EDs on cardiac receptors

Considering the similarities between the actions of the molecules of the EDs and the cocaine, in this study we want to underline the possibility of findings the typical cocaine’s “myocardial remodelling” and pathophysiological effect in the chronic EDs users (Fig. 1).

## Materials and Methods

**Fig. 2:**
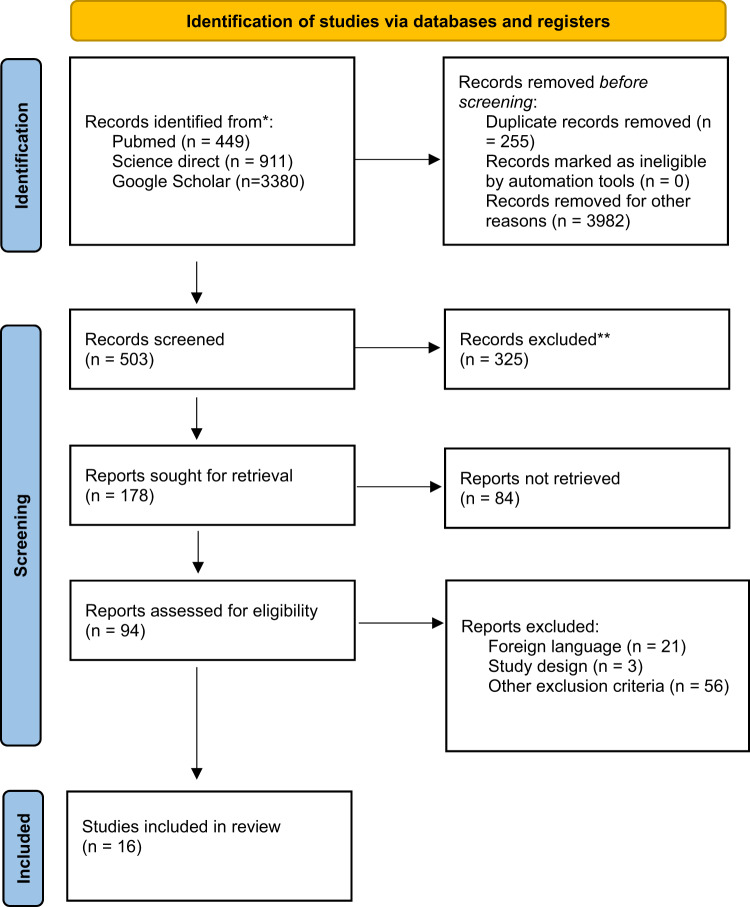
The PRISMA flow diagram of study selection for systematic review

The present systematic review was carried out according to the Preferred Reporting Item for Systematic Review (PRISMA) standards. A methodological appraisal of each study was conducted according to the PRISMA standards, including an evolution of bias. PRISMA 2020 Statement was applied [20]. It consists of a checklist and a flow diagram (Fig. 2).

We performed a review of the English literature regarding the cardiac histopathological patterns associated to the chronic use of EDs. A systematic literature search and critical review of the collected studies were conducted. An electronic search of PubMed (449), Google Scholar (3380) and Science Direct Scopus (911) from the database during the period between 2021 and March 2025 was performed. Databases were investigated using the following research terms “(EDs OR red bull OR monster) AND (heart disease OR cardiac pathophysiology OR cardiac histopathology)”; in all fields [e.g., title, abstract, and keywords].

From this research, a list of abstracts was organized in the form of a dataset, and it was downloaded in a.nbib file and uploaded to Software Zotero 6-0.30, used as a citation manager.

The research group, following a meeting, established the inclusion and exclusion criteria for paper, in accordance with PRISMA standards.

First of all, two investigators (A.G. and M.R.) red all the abstracts found from databases. The bibliographies of all identified papers were examined and cross-referenced to further identify relevant literature. After selecting abstracts and investigating the bibliographies of related papers, data collection began. Two investigators (B.T. and G.D.A.) independently examined papers with titles or abstracts that appeared to be relevant, selecting those that analyzed c cardiac pathophysiology and microscopic patterns of chronic use of EDs.

The data collection process included study selection and data extraction. Disagreements concerning eligibility among the researchers were resolved by consensus. Only papers in English were included.

Data extraction was performed by two investigators (A.G. and M.R.) and verified by additional investigators (A.M., F.D.D. and G.N.).

The current study provides a useful overview for those histopathological patterns associated with chronic use of EDs. However, due to the lack of studies that evidence this condition it is important to continue documenting and sharing such cases while keeping in mind the limitations of this type of research.

## Results

### Human Clinical Outcomes

The analysis of the physio-pathological aspects shows that EDs have been increasingly associated with a range of adverse cardiovascular effects, some of which may be serious or even fatal. These beverages, often containing high levels of caffeine along with other stimulants such as taurine and guarana, can influence both cardiac conduction and myocardial repolarization.

One of the most consistently reported changes is **QTc interval prolongation**, which appears to be **dose dependent**. This prolongation can predispose individuals to potentially life-threatening arrhythmias, especially in susceptible populations such as patients with **familial long QT syndrome (LQTS)**. These individuals are already at higher risk for arrhythmias and sudden cardiac events compared to the general population, and the added stimulant effect of EDs may exacerbate their condition [21, 22].

Documented cardiovascular events linked to ED consumption include **ventricular fibrillation**, even in **otherwise healthy young individuals**. For instance, a case was reported of a 28-year-old man who suffered ventricular fibrillation after excessive ED intake and required hospitalization, although he was ultimately discharged in good health after 6 days [23].

Another striking case involves **reverse Takotsubo cardiomyopathy**, a rare and acute form of stress-induced heart dysfunction, which has been associated with ED consumption [24].

There have also been reports of **acute thrombosis of the left main coronary artery** occurring shortly after consuming an ED, indicating a potential prothrombotic or vasoconstrictive effect of the ingredients [25].

Beyond arrhythmias, EDs have been linked to **alterations in atrioventricular conduction** and **ventricular repolarization**, which may manifest clinically as both **supraventricular and ventricular arrhythmias**, including **atrial fibrillation** [9].

AF, although more common in older populations or those with structural heart disease, has been observed even in younger individuals after consuming high amounts of these beverages.

In terms of hemodynamic effects, studies have shown a **significant and sustained increase in systolic blood pressure** up to 6 h after ED consumption, which surpasses the effects seen with caffeine alone [26].

This hypertensive response could potentially contribute to other complications, such as **subacute aortic dissection**, which has also been documented following ED intake [27]. There are also case reports describing **resuscitated cardiac arrest** [28] and **ST-elevation myocardial infarction (STEMI)** [29], and **sudden cardiac death** following the use of EDs.

Overall, while EDs are often marketed as harmless performance enhancers, the accumulating evidence suggests they may pose **significant cardiovascular risks**, especially when consumed in large quantities or by individuals with underlying cardiac vulnerabilities. Greater awareness and regulation may be needed to mitigate these potential dangers.

### Animal/Preclinical Histopathology

The analysis of cardiac histopathological patterns is currently underrepresented in the literature, with existing evidence primarily originating from studies conducted on animal models. According to these articles, Salih et al. analysed histological patterns of “rabbit” cardiac tissue following Red Bull ED consumption revealed a range of structural alterations consistent with myocardial damage. **Mild muscle necrosis** was observed, often accompanied by **eosinophilic infiltration**, indicating an early inflammatory response. Fragmentation of myocardial fibers and mild necrosis were noted, along with interstitial edema. **Purkinje fibers appeared enlarged**, with distinct rectangular-shaped nuclei. In areas of more advanced damage, moderate muscle necrosis was present, characterized by small, dark pyknotic nuclei, marked hypereosinophilia, and evidence of sarcoplasmic coagulation and clamping. The **normal alignment of myofibers was frequently disrupted** by the accumulation of edematous fluid, and darker red-staining myocytes indicated necrotic regions. Further signs of degeneration included loss of cross striations and fragmentation of cardiac rhabdomyocytes, reflecting progressive deterioration of myocardial integrity following ED exposure [30].

Munteanu et al. conducted a study to assess the effects of different treatments on cardiac morphology in male Wistar rats, stratifying them into distinct experimental groups. In the group administered Red Bull, **intermyofibrillar spaces were notably enlarged** and exhibited extensive **areas of lysis**. Numerous **mitochondria showed altered cristae**, with several demonstrating signs of structural disruption. In the group receiving both Red Bull and ethanol, **intercalated discs (IDs) appeared disorganized**, and a large number of mitochondria displayed severely disrupted cristae. Moreover, this group exhibited an abundance of vesicular structures within the intermyofibrillar spaces, seemingly containing glycogen, along with mitochondria exhibiting complete cristae degradation and further disorganization of the intercalated discs [31].

Demirel et al. conducted a study to evaluate the effects of various treatments on cardiac morphology in Wistar Albino rats. In the group administered EDs, cardiac muscle cells with **cytoplasmic damage** were observed in certain regions. Additionally, eosinophilic cardiac muscle fibers were occasionally detected in this group. In the group receiving a combination of Red Bull and alcohol (RA), extensive **inflammatory cell infiltration** and widespread damage to cardiac muscle cells were noted. **Abnormal morphology of the vascular endothelium** was also observed in specific areas of the heart wall. Based on the total histopathological score, cardiac damage was significantly more pronounced in the RA group compared to the control group. Only a few damaged fibers were found in the alcohol and ED groups, whereas the RA group exhibited damaged muscle fibers in several regions of the striated muscle tissue. Overall, rats exposed to both EDs and alcohol showed a higher degree of cellular damage and more pronounced structural abnormalities in both cardiac and striated muscle tissues [32].

The evidence presented across the reviewed studies consistently indicates that the consumption of EDs, is associated with significant histopathological alterations in cardiac tissue, at least in the examined animal models. Findings from Salih et al., Munteanu et al., and Demirel et al. reveal a common pattern of myocardial damage characterized by muscle fiber necrosis, interstitial edema, disorganization of myocardial architecture, mitochondrial structural abnormalities, and inflammatory infiltration. These effects appear to be exacerbated when EDs are consumed in combination with alcohol, suggesting a synergistic cardiotoxic interaction. Observations such as eosinophilic muscle fibers, disruption of intercalated discs, and severe mitochondrial degradation point to a progressive degenerative process that compromises cardiac tissue integrity. In conclusion, while these findings are based on animal studies, they strongly highlight the potential cardiotoxicity of EDs, especially when combined with alcohol, and emphasize the need for further clinical research in humans to more accurately assess the associated risks (Table 1).

**Table 1:** Cardiac pathophysiology and histology related to energy drinks

References	Animal/person (*n*.)	Age	Pathophysiology	Histology	Ingredients	Dosage and frequency of use
Ciliberti et al. (2025) [9]	–		Hypertension – QTc prolongation – ischemic cardiomyopathy	–	Red bull^a^ Monster^a^ Burn^a^	–
Demirel et al. (2023) [32]	40 Wistar Albino rats divided into 4 groups (energy drink − alcohol − ed + alcohol − control)	–	–	Cardiac muscle cells with damaged cytoplasm − eosinophilic heart muscle fibers [energy drink]/inflammatory cell infiltrations and a large number of damages to cardiac muscle cells were observed—abnormal morphology was observed in the vascular endothelium in some parts of the heart walls [energy drink + ethanol]	Red bull^a^	1.5 mL/100 g ED (Red Bull)
Shah et al. (2019) [33]	34 divided into 3 groups (energy drink A − energy drink B − control)	22.1 years	Prolong the QTc interval and raise blood pressure		Red bull^a^	caffeine (304–320 mg/32-fl oz)
Salih et al. (2018) [30]	30 Males albino rabbits divided into 3 groups (high dose red bull − low dose redbull − control)	–	–	Mild muscle necrosis with eosinophil infiltration—fragmentation with mild myocardial necrosis—edema of Purkinje fibers show enlarged with rectangular nuclei—moderate muscle necrosis—small dark pyknotic nuclei and hypereosinophilia, coagulation, and clamping of the sarcoplasm—the arrangement of myofibers is disrupted by edematous fluid, and the darker red myocytes are necrotic—myocardial fiber degeneration and necrosis with loss of cross striations and fragmentation of cardiac rhabdomyocytes	Red bull^a^	The Group A (high dosage) group contain ten rabbits, all of them administrated with Red Bull (10 mL) by gavage needle for 30 days. The other group (Group B) (low dosage) contains ten rabbits administrated with Red Bull (5 mL) by gavage needle for 30 days.
Munteanu et al. (2018) [31]	Male Wistar rats (28 in 4 groups of 7: control—ethanol—redbull–ethanol + red bull)	–	–	Intermyofibrillar spaces are enlarged, with numerous lysis areas—numerous mitochondria have altered cristae [redbull]/disorganized intercalated disks—numerous mitochondria with disrupted cristae—numerous vesicles that seem to be filled with glycogen [redbull and ethanol]	Red bull^a^	RB group were orally administrated with 1.5 mL/100 g body weight of Red Bull in drinking water daily, for 30 days
Gharacholou et al. (2017) [29]	1 Male	27 years	ST—segment elevation myocardial infarction and normal coronary arteries after consuming energy drinks	–	Rockstar^b^	(Rockstar, Rockstar, Inc., Las Vegas, NV), sometimes 4–5 beverages in a 12-h period, in order to stay awake during his evening work shift in a warehouse
Fletcher et al. (2017) [26]	18 (6 F–12 M)	18–40 years	Prolong the QTc interval and raise blood pressure	–	Commercially EDs (containing 108 g of sugar, vitamin B2, vitamin B3, vitamin B6, and vitamin B12, and a proprietary energy blend of taurine, panax ginseng extract, l-carnitine, caffeine [320 mg], glucuronolactone, inositol, guarana extract, and maltodextrin)	Participants were assigned to consume either a 1-time 32-ounce (946 mL) dose of a commercially available energy drink
Gray et al. (2017) [21]	24 (11 M–13 F)	16–50 years	Acute increase in blood pressure	–	Red bull^a^	2 × RedBullSugar-free cans = TOTAL 160 mg caffeine + 2000 mg taurine in 500 mL
Khan et al. (2015) [28]	1 Male	27 years	Ventricular Fibrillation and Cardiac Arrest	–	Red bull^a^	His ongoing red bull consumption has been almost daily for over 6–8 months
Shah et al. (2014) [22]	1 Male	31 years	QTc prolongation	–	Monster^a^	Two 16-ounce cans (1 L total) of Monster Energy Drink^®^ (providing a total of 320 mg of caffeine) consumed over 45 min
Avci et al. (2013) [34]	1 Male	28 years	Death from sudden cardiac arrest—QTc prolongation	–	Commercially EDs	The patient had drunk 3 cans of 250-mL energy drink 5 h before the basketball match
Kaoukis et al. (2012) [24]	1 Male	24 years	Reverse Takotsubo Cardiomyopathy	–	Commercially EDs	Ingestion of small amounts of an energy drink in little cups one after another
Dufendach et al. (2012) [35]	1 Male	12 years	Unmask a characteristic QT/QTc response	–	Commercially EDs	Routinely consumed at least 16 oz of energy drink every other day for 2 weeks
Benjo et al. (2012) [25]	1 Male	24 years	Left main coronary artery acute thrombosis related to energy drink intake	–	Commercially EDs + vodka	3 drinks of vodka mixed with an energy drink at a local party
Berger et al. (2009) [23]	1 Male	28 years	Resuscitated cardiac arrest with anteroseptal ST Elevation Myocardial Infarction (STEMI)	–	Commercially EDs	Consumed 7–8 cans of a caffeinated “energy drink” between 8 am and his collapse 7 h later
Terlizzi et al. (2008) [36]	1 Female	16 years	Reversible postural tachycardia syndrome	–	Red bull^a^	Started drink Red Bull (4–5 cans a day) 1 week before the onset of orthostatic symptoms

^a^Red Bull (caffeine 80 mg, taurine 1000 mg, glucuronolactone 600 mg, carboidrats 27350 mg, niacin 20 mg, Vitamine B6 10 mg, Vitamine B12 5 µg)—Monster (caffeine 80 mg, taurine 1000 mg, glucuronolactone ND, carboidrats 23500 mg, niacin 21 mg, Vitamine B6 2 mg, Vitamine B12 6 µg)—Burn (caffeine 80 mg, taurine 1000 mg, glucuronolactone 600 mg, carboidrats 36000 mg, niacin 15 mg, Vitamine B6 1.75 mg, Vitamine B12 0.75 µg)

^b^Rockstar Energy Drink (sugar about 63 g, taurine around 1,000 mg, caffeine approximately 160 mg, sodium citrate as an acidity regulator, and caramel color. glucuronolactone about 50 mg, sodium benzoate as a preservative, inositol roughly 25 mg, l-carnitine, guarana seed extract as a natural caffeine source, B-vitamins (niacinamide/B3, pyridoxine/B6, riboflavin/B2, and cyanocobalamin/B12), Panax ginseng root extract, milk thistle extract, and Yellow 5 as a coloring agent)

## Discussion

Cocaine and energy drinks, though pharmacologically and socially distinct, exhibit a range of converging effects on the cardiovascular system, often through overlapping molecular and physiological pathways. Both substances have been associated with acute and chronic cardiotoxicity, driven by alterations in electrophysiological balance, vascular tone, and hemostatic function.

One of the most striking similarities lies in their impact on voltage-gated ion channels, the blockade of ion channels by cocaine represents one of the main mechanisms of its toxicity. Specifically, voltage-gated sodium and potassium channels are involved and inhibited. This leads to electrical disturbances in the heart, increasing the predisposition to the development of arrhythmias, as demonstrated by numerous studies [16, 37]. Cocaine inhibits sodium and potassium channels, disrupting normal cardiac conduction and mimicking the pharmacodynamic profile of class I and III antiarrhythmic agents. This blockade prolongs the myocardial action potential, contributing to QT interval prolongation and increased vulnerability to malignant arrhythmias such as ventricular tachycardia and fibrillation. Moreover, it is associated with additional proarrhythmic factors include sympathetic activation and hemodynamic stress, particularly in predisposed individuals [18]. Furthermore, it has been specifically reported that acute cocaine administration causes rhythm disturbances in a dose-dependent manner. In particular, it induces tachycardia at low doses and bradycardia at higher doses [38].

Similarly, caffeine and taurine—key components of energy drinks— also act on voltage-gated ion channels, particularly the potassium channel KCNH2 (also known as hERG), which plays a critical role in cardiac repolarization. The resulting QTc prolongation and altered myocardial excitability heighten the risk of arrhythmogenesis further elevated in individuals with latent genetic channelopathies [33, 39].

Beyond direct electrophysiological interference, both cocaine and energy drinks exert profound effects on the autonomic nervous system. Cocaine induces intense sympathetic activation through inhibition of norepinephrine reuptake, leading to tachycardia, hypertension, and vasoconstriction. Caffeine can therefore cause extrasystoles, atrial fibrillation, and tachyarrhythmias through a mechanism involving increased myocardial excitability. Taurine leads to magnesium depletion and consequent modulation of electrolyte balance. This enhances the dispersion of repolarization and promotes the onset of malignant arrhythmias [40]. As with cocaine, energy drinks can also affect arterial vascular tone. Sympathetic hyperactivation and endothelial dysfunction can lead to coronary vasoconstriction, causing subclinical ischemia and myocardial infarction. Vasoconstriction induced by caffeine and other stimulants (such as guarana and theophylline) may trigger coronary artery spasm, resulting in subclinical ischemia or myocardial infarction. The underlying mechanisms include sympathetic overactivation, endothelial dysfunction, and increased myocardial oxygen demand [23].

Moreover, both agents have been implicated in endothelial dysfunction and vascular injury. Cocaine impairs endothelial nitric oxide production and promotes oxidative stress, contributing to reduced vasodilatory capacity and enhanced vasoreactivity. Caffeine, while generally considered less potent, can also induce endothelial dysfunction, particularly when consumed in high doses or in combination with other stimulants. This impairment of endothelial function not only compromises coronary perfusion but also sets the stage for thrombotic complications.

Prothrombotic states are another shared consequence. Cocaine has been shown to increase coagulation factors such as fibrinogen and to activate the coagulation cascade, fostering the formation of intravascular thrombi. All these phenomena contribute to a prothrombotic state, predisposing to the formation of intravascular thrombi, which can significantly raise the risk of acute myocardial infarction through a coronary occlusion mechanism driven by thrombosis [41]. In parallel, caffeine, in particular, promotes a pro-inflammatory and pro-thrombotic state by altering nitric oxide production and impairing endothelium-dependent vasodilation [38].

On a structural level, chronic exposure to either substance may lead to myocardial remodelling and dysfunction. Repeated cocaine use is linked to myocardial apoptosis, mitochondrial dysfunction, and direct myocyte necrosis, culminating in dilated cardiomyopathy [4, 42], ventricular remodelling, and heart failure frequently identified in the heart analysed; in fact on autopsy, in subjects who died from chronic cocaine abuse, is typically reported interstitial fibrosis, myofibrillar degeneration, and inflammatory infiltrates of the heart [42, 43]. Although less well-documented, high and chronic intake of energy drinks—especially in combination with alcohol or other stimulants—has also been associated with cardiac remodelling and impaired systolic function in case reports and animal studies.

**Fig. 3:**
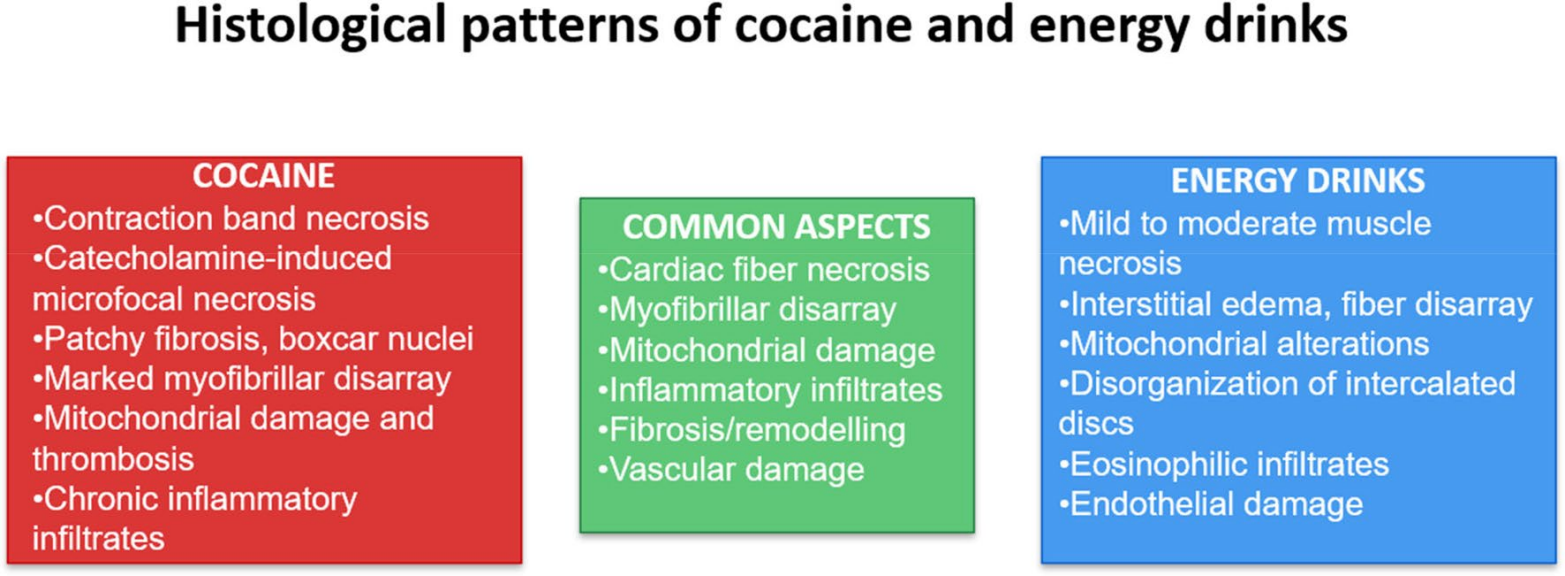
Histological patterns of cocaine and energy drinks

Importantly, the dose-response relationship and context of use must be considered when comparing these substances. Cocaine use is typically associated with higher cardiovascular risk due to its potency and acute pharmacological profile, often in the context of illicit use and polysubstance abuse. In contrast, energy drinks are legally consumed and widely marketed, particularly among adolescents and young adults. Despite their lower individual potency, the high-frequency and large-volume consumption of these beverages—especially during physical activity or in combination with alcohol—can mimic some of the cardiovascular risks observed with cocaine (Fig. 3, Table 2).

**Table 2:** Comparison between energy drinks and cocaine

	Energy drinks	Cocaine
Pathophysiology	QTc interval prolongation Ventricular fibrillation Reverse Takotsubo cardiomyopathy Acute thrombosis of the left main coronary artery Alterations in atrioventricular conduction Supraventricular and ventricular arrhythmia Atrial fibrillation Significant and sustained increase in systolic blood pressure Resuscitated cardiac arrest ST-elevation myocardial infarction Sudden cardiac death	QT interval prolongation malignant arrhythmias Ventricular tachycardia and fibrillation Tachycardia at low doses and bradycardia at higher doses Hypertension Vasoconstriction Coronary vasoconstriction, causing subclinical ischemia and myocardial infarction Coronary artery spasm Fostering the formation of intravascular thrombi Dilated cardiomyopathy
Histology	Mild muscle necrosis/contraction band necrosis (CBN)/microfocal myocardial necrosis Eosinophilic infiltration Purkinje fibers appeared enlarged Normal alignment of myofibers was frequently disrupted/patchy myocardial Intermyofibrillar spaces were notably enlarged Areas of lysis Mitochondria showed altered cristae Intercalated discs (IDs) appeared disorganized Cytoplasmic damage Inflammatory cell infiltration Abnormal morphology of the vascular endothelium	Contraction band necrosis (CBN) Microfocal myocardial necrosis Patchy myocardial fibrosis Cardiomyocytes typically appear thickened, with “boxcar” (squared off) nuclei Myofiber disarray

## Conclusion

In conclusion, although cocaine and energy drinks differ markedly in terms of their legal status, public perception, and pharmacological classification, they share several key pathophysiological mechanisms that adversely affect the cardiovascular system. Both can provoke arrhythmias, impair coronary perfusion, promote thrombosis, and, in chronic contexts, contribute to structural heart disease. This review highlights these parallels to emphasize that, while energy drinks are legal and widely consumed, they may pose a significant public health risk, exhibiting cardiovascular effects reminiscent of illicit stimulants. These findings underscore the need for increased clinical awareness and targeted public education regarding the potential dangers associated with energy drink consumption, supporting a balanced approach to their regulation and public health messaging.

## Data Availability

No datasets were generated or analysed during the current study.

## References

[1] Costantino, A., Maiese, A., Lazzari, J., Casula, C., Turillazzi, E., Frati, P., & Fineschi, V. (2023). The dark side of energy drinks: A comprehensive review of their impact on the human body. *Nutrients,**15*(18), Article 3922. 10.3390/nu15183922 37764707 10.3390/nu15183922 PMC10535526

[2] Higgins, J. P., Babu, K., Deuster, P. A., & Shearer, J. (2018). Energy drinks: A contemporary issues paper. *Current Sports Medicine Reports,**17*(2), 65–72. 10.1249/JSR.0000000000000454 29420350 10.1249/JSR.0000000000000454

[3] Jagim, A. R., Harty, P. S., Tinsley, G. M., Kerksick, C. M., Gonzalez, A. M., Kreider, R. B., Arent, S. M., Jager, R., Smith-Ryan, A. E., Stout, J. R., Campbell, B. I., VanDusseldorp, T., & Antonio, J. (2023). International society of sports nutrition position stand: Energy drinks and energy shots. *Journal of the International Society of Sports Nutrition,**20*(1), Article 2171314. 10.1080/15502783.2023.2171314 36862943 10.1080/15502783.2023.2171314 PMC9987737

[4] Lange, R. A., & Hillis, L. D. (2001). Cardiovascular complications of cocaine use. *New England Journal of Medicine,**345*(5), 351–358. 10.1056/NEJM200108023450507 11484693 10.1056/NEJM200108023450507

[5] Havakuk, O., Rezkalla, S. H., & Kloner, R. A. (2017). The cardiovascular effects of cocaine. *Journal of the American College of Cardiology,**70*(1), 101–113. 10.1016/j.jacc.2017.05.014 28662796 10.1016/j.jacc.2017.05.014

[6] Karch, S. B., & Drummer, O. (2015). *Karch’s pathology of drug abuse* (5th ed.). CRC. 10.1201/b18962

[7] Reissig, C. J., Strain, S., & Griffiths, R. R.Caffeinated energy drinks—a growing problem. *Drug And Alcohol Dependence*. 10.1016/j.drugalcdep.2008.08.001 10.1016/j.drugalcdep.2008.08.001 PMC2735818 18809264

[8] Higgins, J. P., Tuttle, T. D., & Higgins, C. L. (2010). Energy beverages: Content and safety. *Mayo Clinic Proceedings*. 10.4065/mcp.2010.0381 21037046 10.4065/mcp.2010.0381 PMC2966367

[9] Ciliberti, G., Abrignani, M. G., Zilio, F., Temporelli, P. L., Ciccirillo, F., Fortuni, F., Binaghi, G., Iannopollo, G., Cappelletto, C., Albani, S., Maloberti, A., Ceriello, L., Musella, F., Manfredi, R., Scicchitano, P., Riccio, C., Grimaldi, M., Gabrielli, D., Colivicchi, F., Oliva, F., & a nome dell’Area Giovani e dell’Area Cronicità Cardiologica ANMCO. (2024). Substance abuse and cardiovascular risk: Energy drinks. *Giornale Italiano di Cardiologia, 25*(8), 546–556. 10.1714/4309.42924 10.1714/4309.42924 39072593

[10] Jones, G. (2008). Caffeine and other sympathomimetic stimulants: Modes of action and effects on sports performance. *Essays In Biochemistry*. 10.1042/BSE0440109 10.1042/BSE0440109 18384286

[11] Institute of Medicine (US) Committee on Military Nutrition Research. (2001). *Caffeine for the sustainment of mental task performance: Formulations for military operations*. Academies. 25057583

[12] Kong, H., Jones, P. P., Koop, A., Zhang, L., Duff, H. J., & Chen, S. R. W. (2008). Caffeine induces Ca^2+^ release by reducing the threshold for luminal Ca^2+^ activation of the ryanodine receptor. *The Biochemical Journal*. 10.1042/BJ20080489 18518861 10.1042/BJ20080489 PMC2747660

[13] Ellermann, C., Hakenes, T., Wolfes, J., Wegner, F. K., Willy, K., Leitz, P., Rath, B., Eckardt, L., & Frommeyer, G. (2022). Cardiovascular risk of energy drinks: Caffeine and taurine facilitate ventricular arrhythmias in a sensitive whole-heart model. *Journal of Cardiovascular Electrophysiology,**33*(6), 1290–1297. 10.1111/jce.15458 10.1111/jce.15458 35304782

[14] Gutiérrez-Hellín, J., & Varillas-Delgado, D. (2021). Energy drinks and sports performance, cardiovascular risk, and genetic associations; Future prospects. *Nutrients,**13*(3), Article 715. 10.3390/nu13030715 33668219 10.3390/nu13030715 PMC7995988

[15] Grasser, E., Yepuri, G., Dulloo, A., & Montani, J. (2014). Cardiovascular and cerebrovascular effects in response to Red Bull consumption combined with mental stress. *American Journal of Cardiology,**113*(1), 108–112. 10.1016/j.amjcard.2014.10.017 25465941

[16] O’Leary, M. E., & Hancox, J. C. (2010). Role of voltage-gated sodium, potassium and calcium channels in the development of cocaine‐associated cardiac arrhythmias. *British Journal of Clinical Pharmacology*. 10.1111/j.1365-2125.2010.03629.x 20573078 10.1111/j.1365-2125.2010.03629.x PMC2856043

[17] Graziani, M., Antonilli, L., Togna, A. R., Grassi, M., Badiani, A., & Saso, L. (2015). Cardiovascular and hepatic toxicity of cocaine: Potential beneficial effects of modulators of oxidative stress. *Oxidative Medicine and Cellular Longevity*. 10.1155/2016/8408479 26823954 10.1155/2016/8408479 PMC4707355

[18] Zimmerman, J. L. (2012). Cocaine intoxication. *Critical Care Clinics,**28*(4), 517–526. 10.1016/j.ccc.2012.07.003 22998988 10.1016/j.ccc.2012.07.003

[19] Fineschi, V., Baroldi, G., & Silver, M. D. (2006). *Pathology of the heart and sudden death in forensic medicine*. CRC. 10.1201/9781420006438

[20] Page, M. J., McKenzie, J. E., Bossuyt, P. M., Boutron, I., Hoffmann, T. C., Mulrow, C. D., Shamseer, L., Tetzlaff, J. M., Akl, E. A., Brennan, S. E., Chou, R., Glanville, J., Grimshaw, J. M., Hróbjartsson, A., Lalu, M. M., Li, T., Loder, E. W., Mayo-Wilson, E., McDonald, S., …, Moher D. (2021). The PRISMA 2020 statement: An updated guideline for reporting systematic reviews. *Bmj*. 10.1136/bmj.n71 10.1136/bmj.n71 PMC8005924 33782057

[21] Gray, B., Ingles, J., Medi, C., Driscoll, T., & Semsarian, C. (2017). Cardiovascular effects of energy drinks in familial long QT syndrome: A randomized cross-over study. *International Journal of Cardiology,**231*, 150–154. 10.1016/j.ijcard.2016.12.019 28189188 10.1016/j.ijcard.2016.12.019

[22] Shah, S. A., Lacey, C. S., Bergendahl, T., Kolasa, M., & Riddock, I. C. (2014). QTc interval prolongation with high dose energy drink consumption in a healthy volunteer. *International Journal of Cardiology*, *172*(2), e336–337. 10.1016/j.ijcard.2013.12.218 24447738 10.1016/j.ijcard.2013.12.218

[23] Berger, A., & Alford, K. (2009). Cardiac arrest in a young man following excess consumption of caffeinated energy drinks. *Medical Journal of Australia,**190*(1), 41–43. 19120009 10.5694/j.1326-5377.2009.tb02263.x

[24] Kaoukis, A., Panagopoulou, V., Mojibian, H. R., & Jacoby, D. (2012). Reverse Takotsubo cardiomyopathy associated with the consumption of an energy drink. *Circulation*, *125*(12), 1584–1585. 10.1161/CIRCULATIONAHA.111.057505 22451608 10.1161/CIRCULATIONAHA.111.057505

[25] Benjo, A. M., Pineda, A. M., Nascimento, F. O., Zamora, C., Lamas, G. A., & Escolar, E. (2012). Left main coronary artery acute thrombosis related to energy drink intake. *Circulation,**125*(11), 1447–1448. 10.1161/CIRCULATIONAHA.111.086017 22431887 10.1161/CIRCULATIONAHA.111.086017

[26] Fletcher, E. A., Lacey, C. S., Aaron, M., Kolasa, M., Occiano, A., & Shah, S. A. (2017). Randomized controlled trial of high-volume energy drink versus caffeine consumption on ECG and hemodynamic parameters. *Journal of the American Heart Association*. 10.1161/JAHA.116.004448 28446495 10.1161/JAHA.116.004448 PMC5524057

[27] Jonjev, Z. S., & Bala, G. (2013). High-energy drinks may provoke aortic dissection. *Collegium Antropologicum,**37*(Suppl 2), 227–229. 23914511

[28] Khan, R., Osman, M., Zafar, S., & Sen, S. (2015). Energy drink induced ventricular fibrillation and cardiac arrest: A successful outcome. *Journal of Medical Cases,**6*(9), 409–412. 10.14740/jmc2259w

[29] Gharacholou, S. M., Ijioma, N., Banwart, E., & Munoz, F. D. C. (2017). ST-segment elevation myocardial infarction and normal coronary arteries after consuming energy drinks. *Case Rep Cardiol*, *2017*(1), 4061205. 10.1155/2017/4061205 28203465 10.1155/2017/4061205 PMC5288504

[30] Salih, N., Abdul-Sadaand, I., & Abdulrahman, N. (2018). Histopathological effect of energy drinks (Red Bull) on brain, liver, kidney, and heart in rabbits. *Medical Journal of Babylon,**15*(1), Article 16. 10.4103/MJBL.MJBL_5_18

[31] Munteanu, C. (2018). Long-term consumption of energy drinks induces biochemical and ultrastructural alterations in the heart muscle. *The Anatolian Journal of Cardiology*. 10.14744/AnatolJCardiol.2018.90094 29724975 10.14744/AnatolJCardiol.2018.90094 PMC6280269

[32] Demirel, A. (2023). Histopathological changes in the myocardium caused by energy drinks and alcohol in the mid-term and their effects on skeletal muscle following ischemia-reperfusion in a rat model. *Anatolian Journal of Cardiology,**27*(1), 12–18. 10.14744/AnatolJCardiol.2022.2003 36680442 10.14744/AnatolJCardiol.2022.2003 PMC9893703

[33] Shah, S. A., Szeto, A. H., Farewell, R., Shek, A., Fan, D., Quach, K. N., Bhattacharyya, M., Elmiari, J., Chan, W., O’Dell, K., Nguyen, N., McGaughey, T. J., Nasir, J. M., & Kaul, S. (2019). Impact of high volume energy drink consumption on electrocardiographic and blood pressure parameters: A randomized trial. *Journal of the American Heart Association,**8*(11), Article e011318. 10.1161/JAHA.118.011318 PMC6585360 31137991

[34] Avcı, S., Sarıkaya, R., & Büyükcam, F. (2013). Death of a young man after overuse of energy drink. *American Journal of Emergency Medicine,**31*(11), 1624e3-1624e4. 10.1016/j.ajem.2013.06.031 10.1016/j.ajem.2013.06.031 23896014

[35] Dufendach, K. A., Horner, J. M., Cannon, B. C., & Ackerman, M. J. (2012). Congenital type 1 long QT syndrome unmasked by a highly caffeinated energy drink. *Heart Rhythm,**9*(2), 285–288. 10.1016/j.hrthm.2011.10.011 22001708 10.1016/j.hrthm.2011.10.011

[36] Terlizzi, R., Rocchi, C., Serra, M., Solieri, L., & Cortelli, P. (2008). Reversible postural tachycardia syndrome due to inadvertent overuse of Red Bull. *Clinical Autonomic Research,**18*(4), 221–223. 10.1007/s10286-008-0483-y 18682891 10.1007/s10286-008-0483-y

[37] Gartz, A., Pawlik, E., Eckhardt, J., Ritz-Timme, S., Huhn, R., & Mayer, F. P. (2020). Effects of cocaine and levamisole (as adulterant) on the isolated perfused Langendorff heart. *International Journal of Legal Medicine*. 10.1007/s00414-020-02300-5 32377925 10.1007/s00414-020-02300-5 PMC7417403

[38] Mersereau, E., Poitra, S. L., Espinoza, A. M., Crossley, D. A., & Darland, T. (2015). The effects of cocaine on heart rate and electrocardiogram in zebrafish (*Danio rerio*). *Comparative Biochemistry and Physiology, Part C: Toxicology & Pharmacology*. 10.1016/j.cbpc.2015.03.007 10.1016/j.cbpc.2015.03.007 PMC4458413 25847362

[39] Fletcher, E., Lacey, C., Aaron, M., et al. (2017). Randomized controlled trial of an energy drink’s cardiovascular effects in healthy adolescents. *Journal of the American Medical Association*, *318*(3), 308–309.

[40] Goldfarb, M., Tellier, C., & Thanassoulis, G. (2014). Review of published cases of adverse cardiovascular events after ingestion of energy drinks. *American Journal of Cardiology,**113*(1), 168–172. 24176062 10.1016/j.amjcard.2013.08.058

[41] Kugelmass, A. D., Oda, A., Monahan, K., Cabral, C., & Ware, J. A. (1993). Activation of human platelets by cocaine. *Circulation,**88*(3), 876–883. 10.1161/01.CIR.88.3.876 7689042 10.1161/01.cir.88.3.876

[42] Schwartz, B. G., Rezkalla, S., & Kloner, R. A. (2025). Cardiovascular effects of cocaine. *Circulation,**122*(24), 2558–2569. 10.1161/CIRCULATIONAHA.110.940569 10.1161/CIRCULATIONAHA.110.940569 21156654

[43] Bachi, K., Mani, V., Jeyachandran, D., Fayad, Z. A., Goldstein, R. Z., & Alia-Klein, N. (2017). Vascular disease in cocaine addiction. *Atherosclerosis,**262*, 154–162. 10.1016/j.atherosclerosis.2017.03.019 28363516 10.1016/j.atherosclerosis.2017.03.019 PMC5757372

